# A scale of functional divergence for yeast duplicated genes revealed from analysis of the protein-protein interaction network

**DOI:** 10.1186/gb-2004-5-10-r76

**Published:** 2004-09-15

**Authors:** Anaïs Baudot, Bernard Jacq, Christine Brun

**Affiliations:** 1 Laboratoire de Génétique et Physiologie du Développement, IBDM, CNRS INSERM Université de la Méditerranée, Parc Scientifique de Luminy, Case 907, 13288 Marseille Cedex 9, France

## Abstract

**Background:**

Studying the evolution of the function of duplicated genes usually implies an estimation of the extent of functional conservation/divergence between duplicates from comparison of actual sequences. This only reveals the possible molecular function of genes without taking into account their cellular function(s). We took into consideration this latter dimension of gene function to approach the functional evolution of duplicated genes by analyzing the protein-protein interaction network in which their products are involved. For this, we derived a functional classification of the proteins using PRODISTIN, a bioinformatics method allowing comparison of protein function. Our work focused on the duplicated yeast genes, remnants of an ancient whole-genome duplication.

**Results:**

Starting from 4,143 interactions, we analyzed 41 duplicated protein pairs with the PRODISTIN method. We showed that duplicated pairs behaved differently in the classification with respect to their interactors. The different observed behaviors allowed us to propose a functional scale of conservation/divergence for the duplicated genes, based on interaction data. By comparing our results to the functional information carried by GO annotations and sequence comparisons, we showed that the interaction network analysis reveals functional subtleties, which are not discernible by other means. Finally, we interpreted our results in terms of evolutionary scenarios.

**Conclusions:**

Our analysis might provide a new way to analyse the functional evolution of duplicated genes and constitutes the first attempt of protein function evolutionary comparisons based on protein-protein interactions.

## Abstract 2

Protein-protein interaction networks were used to analyze the functional evolution of duplicated genes in yeast. Pairs of paralogs can be grouped into 3 classes, which likely form part of a continuous scale of diversity.

## Background

Complete genome analysis showed the tremendous extent to which gene and genome duplication events have shaped genomes over time. Remarkably, 30% of the *Saccharomyces cerevisae *genome, 40% that of *Drosophila melanogaster*, 50% that of *Caenorhabditis elegans*, and 38% of the human genome are composed of duplicated genes [1,2]. According to Ohno's theory [3], such duplication events should have provided genetic raw material, a source of evolutionary novelties, that could have led to the emergence of new genes and functions through mutations followed by natural selection. But despite the recent increase in genomic knowledge, the patterns by which gene duplications might give rise to new gene functions over the course of evolution remain poorly understood. This is mainly explained by the fact that there are very few ways of experimentally investigating the evolution of function of duplicated genes. Studying the function of duplicated genes usually means estimating the extent of the conservation/divergence between duplicates from comparison of actual sequences. For this purpose, the sequence divergence, the divergence time and the selective constraints on gene pairs are usually calculated (as in [4]). Given that these calculations are only valid on a relatively short timescale [4,5], they exclude *de facto *the study of ancient duplication events (such as the complete duplication of the yeast genome [6-8]), even though remnants of such events are still present in the genomes [9]. Enlarging the timescale on which we are able to work is thus a desirable goal, which may be reached by using other means to evaluate the functional conservation/divergence between duplicates.

In addition, sequence analysis generally only reveal the possible molecular (biochemical) function(s) of proteins and even this only applies when domains of known function are identified in the sequences. As discussed previously [10], the function of a gene or protein can be defined at several integrated levels of complexity (molecular, cellular, tissue, organismal) As far as genome evolution is concerned, consideration of the functional evolution of genes and proteins not only at the basal molecular level, but also at upper, more integrated, levels is particularly important. In this respect, it is essential to consider the cellular function of genes/proteins - that is, the biological processes they are involved in. One can easily imagine, for instance, that the evolution of a duplicated pair of protein kinases, having the same molecular function, could potentially result in the emergence of a new signaling pathway involved in a different cellular function. Being able to study the evolutionary fate of duplicated genes at the level of cellular function using bioinformatics methods, something that was quite difficult until now, may thus provide new insights into the field. To do so, one needs to be able to easily compare the functions of many proteins at once and to estimate their functional similarities at the cellular level.

Function comparison was one of our aims while developing PRODISTIN, a computational method that we recently proposed [11]. This method permits the functional classification of proteins solely on the basis of protein-protein interaction data, independently of sequence data. It clusters proteins with respect to their common interactors and defines classes of proteins found to be involved in the same cellular functions.

In the work presented here, we addressed the question of the cellular functional fate of duplicated genes in the yeast *S. cerevisiae*, focusing on the 899 duplicated genes which represent remnants of an ancient whole-genome duplication (WGD) [6-8]. This event took place 100-150 million years ago in the *Saccharomyces *lineage, after the divergence from *Kluyveromyces waltii*, and was probably followed by a gene-loss event leading to the current *S. cerevisiae *genome [8]. Overall, these duplicated genes form 460 pairs of paralogs, accounting for 16% of the current genome [6].

After applying the PRODISTIN method to the yeast interactome, we established and analyzed the functional classification of the duplicated yeast genes originating from the WGD. This analysis allowed us to compare the cellular function(s) of 41 paralog pairs for which enough interaction data was available. Three different behaviors of the pairs of paralogs in respect of the PRODISTIN classification were identified from this analysis, allowing us to establish a scale of functional divergence for the duplicated genes based on the protein-protein network analysis. This work validates the use of interaction data and the analysis of interaction networks as a new means of investigating evolutionary processes at the level of the cellular function.

## Results

### GO annotations do not functionally distinguish between duplicated pairs from the ancient genome duplication

To obtain a first estimation of the functional conservation/divergence of the yeast duplicated genes, we analyzed available textual information relative to the actual functions of the 460 pairs of paralogs from the WGD. For this purpose, we used the Gene Ontology (GO) annotations. The Gene Ontology consortium [12] develops structured controlled vocabularies describing three aspects of gene function: 'Molecular Function' describes the biochemical function of proteins (their molecular activity); 'Biological Process' describes their cellular function (the "broad biological goals that are accomplished by ordered assemblies of molecular functions"); and 'Cellular Component' describes their subcellular localization. These structured vocabularies, or ontologies, are not organized as hierarchies but as directed acyclic graphs (DAGs), in which child terms (the more specialized terms) can have several parent terms (less specialized terms). These functional annotations thus provide a means of comparing gene functions as long as one is able to take into account the structure of the ontology in the comparison process. We performed a pairwise comparison of the functions of the 460 pairs of duplicates by processing their functional GO annotations with GOproxy [13]. This tool calculates a functional distance between genes based on the shared and specific GO annotations. The calculation is made separately for the three ontologies, and for each gene the complete hierarchy of GO terms, from the root term to the leaf term of the DAG, is considered in the comparison process without differentiating the two parent-child relationships existing in GO (the 'is-a' and the 'has-a' relations) (for details see Materials and methods). Two genes that do not share any GO terms would have a maximum distance value (equal to 1), whereas two genes sharing exactly the same set of GO terms would have a minimum distance value (equal to 0).

**Figure 1:**
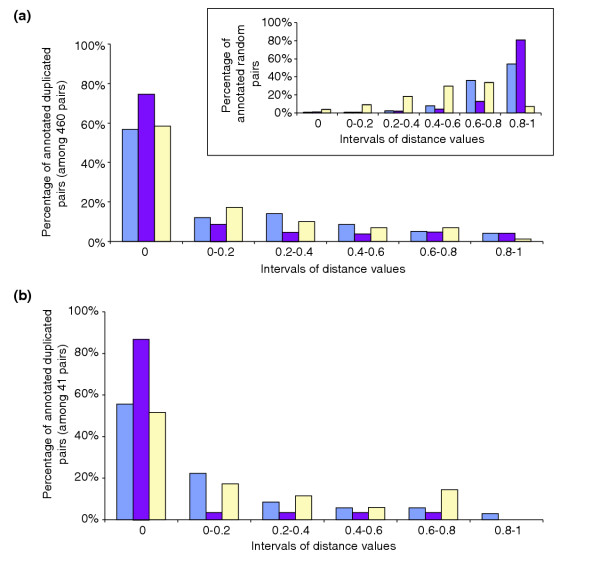
Distribution of functional distances between duplicated pairs based on Gene Ontology annotations. The annnotations are for 'Biological Process' (blue), 'Molecular Function' (purple) and 'Cellular Component' (light yellow). Distributions of distances (ranging from 0 to 1) based on annotations for **(a) **the 460 duplicated pairs, (a, inset) randomly selected pairs and **(b) **the 41 duplicated pairs present in the PRODISTIN tree.

The distributions of the calculated distance values are showed in Figure 1. First, as expected, paralog pairs are globally closer in term of functional distance based on the annotations (Figure 1a) than pairs of proteins chosen randomly from the proteome (Figure 1a, inset). Indeed, the distribution of the distances peaks at the minimum distance value for the paralogs while it peaks at the maximum distance value for the randomly selected pairs.

Second, the vast majority of the duplicated pairs do not differ significantly when Molecular Function terms are compared: 74.5% of the pairs have a zero distance based on annotations (Figure 1a, purple bars). This could be explained by the fact that on one hand, a tight relationship exists between protein sequence similarity and molecular function(s) similarity, and on the other the majority of the paralogs share a percentage sequence identity above the 'twilight zone' (20-35%) [14], usually considered as a threshold for molecular function similarity.

Given that paralogs with the same molecular function may potentially be involved in different cellular functions, we also considered the Biological Process annotations of gene products. Interestingly, the majority of the paralogs also display a zero distance value, suggesting that a majority of duplicated genes from the ancient duplication do not significantly differ when considering the cellular function annotations. However, although the distribution of the distances between the duplicates for the Biological Process annotations displays the same overall shape, only 56.5% of the pairs show a zero value (Figure 1a, blue bars) as compared to 74.5% for the Molecular Function annotations. The fact that, on average, the molecular functions of duplicated pairs are more conserved than their corresponding cellular functions may reflect the fact that changes in function that occurred during evolution are more measurable and discernible at the cellular level than at the molecular level at the present time. This is corroborated by the fact that paralog pairs are found to be globally closer according to the Molecular Function annotation compared to the Biological Process annotation when the expectation values are calculated for each distribution, whereas the converse is encountered for randomly selected pairs (see Additional data file 1). Similarly, changes in subcellular localization (Cellular Component annotations, Figure 1a, yellow bars) also appear to be more apparent than changes in Molecular Function (see Additional data file 1).

### PRODISTIN interaction network analysis: three classification behaviors

Immediately after a genome-duplication event, the two duplicated proteins will have the same interactors. As time goes by and mutations occur, these proteins may gain or lose interactors; that is, the number of interactors for each protein of the pair may change as well as their identity. Taking account of the fact that protein action is seldom isolated but rather is exerted in concert with other proteins, studying duplicates according to the interactors they still share and the ones they have lost or acquired since the duplication event may give a hint about how their cellular functions have evolved.

**Figure 2:**
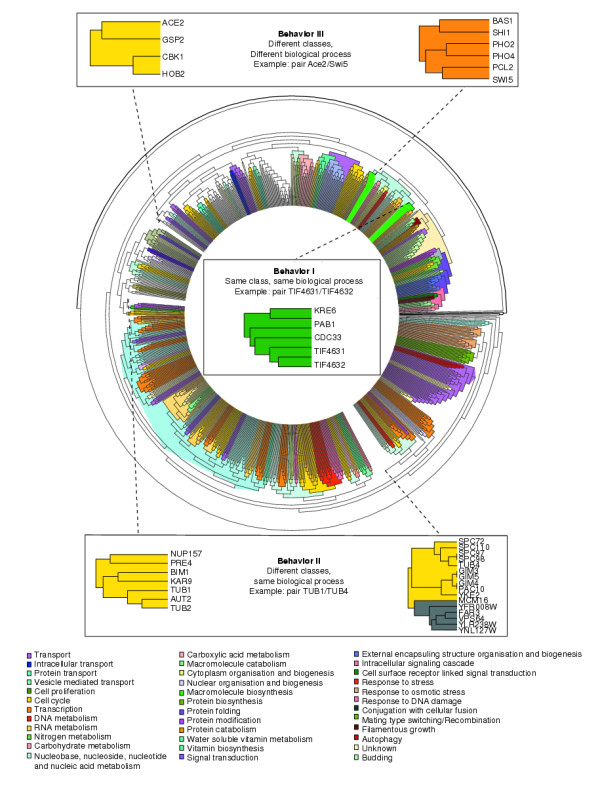
PRODISTIN classification tree for 890 yeast proteins. PRODISTIN classes have been colored according to their corresponding Biological Process annotations. Protein names have been omitted for clarity. The tree contains 41 out of 460 duplicated pairs, the remnant of the ancient whole-genome duplication. Examples of PRODISTIN classes illustrating the three different behaviors of duplicated pairs have been extracted and enlarged from the tree. Their original position in the tree is shown by dashed lines.

We thus applied the PRODISTIN method [11] to 4,143 selected binary protein-protein interactions involving 2,643 yeast proteins. Briefly, the PRODISTIN method consists of three different steps: first, a functional distance is calculated between all possible pairs of proteins in the interaction network with regard to the number of interactors they share (proteins must have at least three interactors to be considered further); second, all distance values are clustered, leading to a classification tree; third, the tree is visualized and subdivided into formal classes. A PRODISTIN class is defined as the largest possible sub-tree composed of at least three proteins sharing the same functional annotation and representing at least 50% of the individual class members for which a functional annotation is available. Classes of proteins are then analyzed for their biological relevance and tested for their statistical robustness (see Materials and methods and [11] for a detailed explanation). The relevance of the method has been assessed biologically and statistically in a previous study (its first application to a smaller interaction dataset led to the prediction of the cellular function of 42 uncharacterized yeast proteins with a success rate of 67% [11]). In the present work, 890 proteins were classified (Figure 2). Among them, 154 correspond to products of duplicated genes from the ancient duplication and 82/154 form 41 pairs of paralogs. These 41 pairs thus correspond to the only pairs from the ancient duplication for which more than three interaction partners per protein are presently known. Then, following the PRODISTIN procedure, the clustering of the proteins was analyzed, defining classes of proteins involved in the same cellular function(s) according to the GO Biological Process ontology (for details, see Materials and methods). In total, 123 classes corresponding to 53 different cellular functions were identified in the tree (see Additional data file 2) and evaluated statistically (data not shown), allowing the classification of 38/41 pairs of duplicated genes (Table 1).

We then investigated the details of the distribution of the duplicates in the tree by analyzing the PRODISTIN classes. Interestingly enough, three different situations were encountered (Figure 2, Table 1). First, for 26 pairs both gene products were found in the same class. This means that their list of interactors is very similar and that these proteins should thus be involved in the same biological process. This is illustrated by Tif4631 and Tif4632 (Figure 2), which are subunits of the translation initiation complex that binds the cap on the 5' end of mRNAs [15]. In our analysis they both belong to a class devoted to 'Protein biosynthesis'. Interestingly, they are clustered with other actors of the initiation of translation (Cdc33, Pab1), as well as with proteins involved in cell-wall biogenesis (Kre6, Pkc1, Stt3), thus reinforcing the recent proposal of the existence of a functional link between these two biological processes [16].

Second, three other pairs of duplicates were recovered in different PRODISTIN classes, relatively far away when considering the tree topology (they therefore no longer share the majority of their interactors), but interestingly, the classes containing the duplicates were dedicated to the same biological process. This is reminiscent of a previous observation we made while studying in detail the rationale sustaining the PRODISTIN clustering [11]: classes distant in the tree but corresponding to the grouping of proteins involved in the same biological process often correspond to different aspects of the same biological process. This is the case for the pair composed of Tub1 and Tub4 (Figure 2), which are classified in different PRODISTIN classes both annotated 'cytoplasm organization and biogenesis' and 'cell cycle' (PRODISTIN classes may be annotated with several cellular functions [11]). These two proteins are structural components of the cytoskeleton that are implicated in microtubule organization. But strikingly, these two paralogous genes have different roles relative to microtubules. Tub1 is an alpha-tubulin and thus a component of the microtubule itself, whereas Tub4 is a gamma-tubulin involved in the nucleation of the microtubules on both the nuclear and the cytoplasmic sides of the spindle-pole body [17]. Consequently, the class containing Tub1 is more structural and mainly composed of proteins implicated in microtubule formation, orientation and catabolism (Kar9, Bim1, Pre4), whereas the class containing Tub4 includes actors of the nuclear processes in which the microtubules are involved: chromosome segregation, spindle orientation and nuclear migration (Spc72, Spc97, Spc98, Spc110, Mcm16, Yfr008w, Far3, Vps64, Ylr238w, Ynl127w). Thus, it appears that the PRODISTIN classification of these two paralogous proteins reflects their functions in two different aspects of the same biological process.

Finally, nine pairs of duplicated genes were found in different classes devoted to different biological processes. This is exemplified by the case of Ace2 and Swi5 (Figure 2), which are two transcription factors regulating the expression of cell-cycle-specific genes. Although they regulate a shared set of genes *in vivo*, they display different specificities in some cases. Swi5 specifically promotes transcription of the *HO *gene whereas Ace2 localizes to daughter cell nuclei after cytokinesis, regulates the expression of daughter-specific genes and delays the G1 progression in daughters [18-20]. The PRODISTIN classification was successful in pointing towards these differences as Swi5 and Ace2 localize in different classes annotated for 'transcription' and 'cell cycle', respectively. Indeed, Swi5 is found with Pho2, a transcription factor acting in a combinatorial manner, with which it interacts to regulate *HO *transcription [21]. Other Pho2 partners populate the rest of the class. On the other hand, Ace2 partitioned with Mob2 and Cbk1, which form a kinase complex regulating the localization of Ace2 in the daughter cell [20].

Overall, this analysis shows that the duplicated gene pairs from the ancient duplication present in the tree display three different behaviors in respect of the PRODISTIN classification (Table 2). The three groups are populated differently: 63% of the protein pairs are located in the same class, and are therefore involved in the same biological process (behavior I); 7.5% of the duplicated pairs are located in different classes with the same function, therefore suggesting that they are involved in different aspects of the same biological process (behavior II); and, finally, the remaining 22% are implicated in different cellular functions because they are located in different classes devoted to different biological processes (behavior III).

We propose considering the three behaviors identified by the PRODISTIN classification as a scale of functional divergence for duplicated pairs. First, the duplicated pairs found in the same class and which essentially have identical interactors would compose the basic level of the scale. This level represents paralogous genes for which cellular function is identical or highly conserved. Higher in the functional scale of divergence are found the duplicates that have different interactors. They are found either in different classes of the same cellular function, thus defining the intermediate level of the functional scale of divergence, or in different classes of different function. This latter case populates the higher level of the scale and represents paralogs for which the cellular function has diverged.

### The relationship between the functional distance based on annotation and the classification behavior based on protein-protein interactions

**Figure 3:**
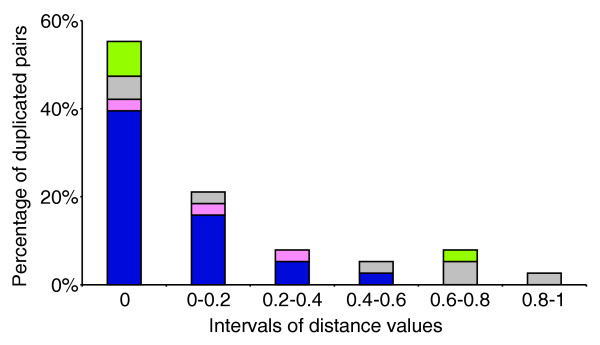
Repartition of the 3 different PRODISTIN behaviors in respect to the distribution of the GO-based functional distances (ranging from 0 to 1) between the 41 duplicated pairs. Behaviors are classified as: same class, same function (behavior I, blue); different classes, same function (behavior II, pink); different classes, different functions (behavior III, gray); not classified (green). Results are shown for the Biological Process annotations only.

As noted above, most of the 460 duplicated gene pairs from the ancient duplication were not distinguishable when considering either the functional annotations for Molecular Function or Biological Process as their functional distances based on annotations were mainly equal or close to zero. We have also shown (Figure 1b) that the subset of 41 paralogous pairs characterized in the PRODISTIN analysis exhibits the same distribution of distance values based on annotations as the 460 pairs. Because the PRODISTIN method allowed us to distinguish three categories of duplicated gene pairs with different types of functional similarities, we wondered if and how the results of the annotation and interaction clustering were correlated. To investigate this, we reported the PRODISTIN behaviours of the paralogs on the distribution of their functional distance based on the Biological Process annotations (Figure 3). Among the duplicated pairs that are similarly annotated, we were able not only to distinguish gene pairs found in the same class, as expected for a correlation between the results of the two approaches (behavior I, blue), but also gene pairs involved in different aspects of the same biological process (behavior II, pink) as well as gene pairs not implicated in the same biological processes (behavior III, gray). The last two cases reveal that whereas annotations do not allow us to differentiate certain paralogs from each other functionally, interactions do unveil subtle functional differences. Conversely, paralogous genes may be grouped in the same PRODISTIN class even though their annotations are not completely similar (up to an annotation-based functional distance equal to 0.6). Interestingly, pairs of duplicated genes partitioning into different classes with different functions are encountered independently of the functional distance based on annotation range. This again underlines the fact that the classification based on interactions identifies functional details that are not discernible at the level of annotation only. Therefore, the protein-protein interactions processed by PRODISTIN bring supplementary functional information about the function of the duplicated genes.

### Sequence evolution versus functional evolution of duplicated genes

**Figure 4:**
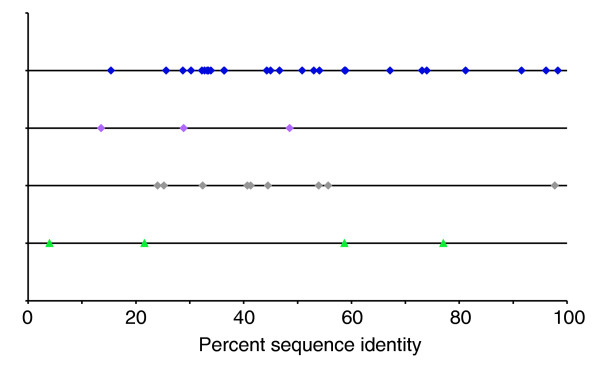
Percent of sequence identity between the 41 duplicated protein pairs. Proteins were classified as belonging to the same class (blue diamonds), different classes with the same function (pink diamonds), different classes with different functions (gray diamonds), or not classified (green triangles).

The availability of 41 yeast paralog pairs for which a pairwise functional comparison can be proposed, offers for the first time the possibility of studying the relationship (if any) between sequence conservation/divergence and evolution of cellular function. Because we have proposed here a three-level scale of possible functional divergence between paralog pairs, what can be said about the sequence-identity patterns shown by protein pairs within and between these three groups? To answer this question, 41 binary sequence comparison analyses were performed (one for each paralogue pair) and the results are displayed according to the classification behavior of the pair identified in the PRODISTIN analysis (Figure 4). If paralogs displaying behaviors I, II and III are compared, three observations can be made: first, all gene pairs that show more than 55% sequence identity display behavior I, with one noticeable exception. It is clear, however, that despite the fact that all the protein pairs of this class have been classified by the PRODISTIN analysis as essentially having a conserved function, their degree of sequence identity covers, in a nearly uniform manner, a wide range comprising 16 to 95% sequence identity. Second, and conversely, gene pairs with between 15 and 55% sequence identity are found in all three classes, clearly indicating that neither cellular functional similarity nor divergence can confidently be deduced for paralog pairs with sequence identity falling in this range. Third and strikingly, no clear distinction can be made on the basis of sequence identity between paralogs found in different classes with (behavior II) or without (behavior III) identical functions. In summary, as suggested by a preliminary study [22], a simple relationship cannot be established between sequence identity and the cellular functional similarity revealed by the interaction-network analysis. So, as previously shown for the annotations, the functional classification based on interactions is able to underline properties of the duplicates that are not discernible when only sequences are compared.

## Discussion

### Bioinformatic study of the interaction network as a tool to investigate the function of the duplicated genes

We have shown here that studying the cellular interactome using bioinformatics methods leads to a proposal of a functional scale of divergence for yeast duplicated genes. As our work makes use of functional gene annotations and interaction lists, it is important to examine how the quality of these two types of data could potentially affect the conclusions that can be drawn from our studies.

Gene annotations provided by the GO consortium [12] are the result of collaborative work by experts, and all annotations are supported by at least one type of experimental evidence. This, together with the use of a controlled vocabulary consistently applied for all annotations, is in principle a good guarantee of annotation quality. However, several potential problems should be taken into account when using annotations. First, all gene products are not annotated. This is the case for 30% of the pairs of duplicated genes, for which at least one gene is not annotated. Second, annotation errors can propagate in the databases, due to the transfer of annotations from gene to gene based only on sequence or structural similarities. In GO, some functional annotations are "inferred from sequence or structural similarity" (ISS), meaning that the annotation assignment is not supported by experimental evidence *per se*. It can then can be argued that paralog pairs may be more prone to such annotation transfers than other genes because of their sequence identity. In such a case, our measure of functional distance according to annotations would be largely meaningless. We thus estimated the amount of genes for which GO annotations are solely 'inferred from sequence or structural similarity'. Interestingly enough, they account, at the level of the complete genome, for only 10.3% and 4.95% of the Molecular Function and the Biological Process annotations, respectively. Similar low values are encountered for the 460 pairs of paralogs (11.2% and 4.5%), allowing us to neglect the weight of such inferred annotations in our distance calculation.

As far as the quality of interactions is concerned, two main problems result from erroneous (false-positive) interactions and missing (false-negative) interactions. Taking into account that the PRODISTIN method was largely statistically assessed for robustness against the presence of false interactions in our previous study [11], we can anticipate that the classification behaviors found in the present analysis will be confirmed, or only slightly modified, in the near future when new interactions are discovered.

### The ancestral yeast genome duplication as a case study for functional evolution of paralogs

In the present analysis, we worked solely on pairs of paralogs that supposedly originated from the ancient WGD [6,7]. This choice was made for several reasons. First, after the yeast WGD hypothesis, we can consider that all genes, remnants from this event, have duplicated simultaneously. This sets a 'time 0' for the duplication event and therefore enables us to avoid the problem of determining the age of the duplication events, a problem inherent in all genome-wide analyses of paralogs. Second, after a WGD, polyploidization preserves the necessary stoechiometric relationships between gene products, while the duplication of a single gene does not: duplicates are then out of balance with their interacting partners. This is an important parameter to consider when one wants to study the evolution of the duplicated genes through the analysis of interactions, as we did in this work. Third, studying the remnants of a WGD after more than 100 million years [7,23] allows one to estimate how the sequence, function and interactors of the paralog gene products have evolved since their origin, when their sequence, function(s) and interactor(s) were identical.

An important issue for the interpretation of our results is the validity of the hypothesis of the existence of a WGD in *S. cerevisiae*. Initially proposed by Wolfe and Shields [7], the WGD model has been controversial and alternative models of local duplications have been proposed [24-27]. Very recently, a novel proof of WGD was provided [8]. Among the 460 paralog pairs we studied, 362 were shown by this new analysis to arise from the WGD. Revisiting our results to take into account the new dataset of duplicated genes did not change them drastically. The distribution of the duplicated pairs becomes 68, 4.5 and 18% for the three different categories of classification behaviors (I, II, III), respectively, compared to 63, 7.5 and 22% for the dataset we used (Table 2).

### The evolution of cellular function: from the scale of functional divergence to the evolutionary fates of the duplicated genes

Our study was driven by the idea that investigating the cellular rather than the molecular function of the duplicated genes might provide new information about the extent of their actual divergence and, consequently, might help us to envisage how their cellular function has evolved since the duplication event. Indeed, the first important outcome of our study, based on the comparison of annotations for duplicated pairs, is that although both the molecular and cellular functions of the majority of protein pairs have been conserved since the date of the WGD, cellular functions have evolved more rapidly than molecular functions. Although this finding could seem rather intuitive, it is, to the best of our knowledge, the first time that evidence has been proposed in its favor. Conservation of the same molecular function for two duplicated proteins while allowing the diversification of their cellular functions may represent a simple and economical way of introducing functional diversity and complexity in a controlled manner during evolution. This may be the result of a change in interaction partners and/or subcellular localization.

The second important result of our study is that since the date of the ancient WGD, cellular functions have evolved at variable rates, since a scale of functional divergence can be detected. In this respect, we propose to interpret this functional scale of divergence in the light of different theoritical evolutionary scenarios for cellular function.

First, the first level of the functional scale (behavior I) may contain duplicates which have been conserved as such, because keeping two copies may confer an evolutionary benefit on the cell (for instance, Rps26A/Rps26B; Table 1).

Second, we propose that the majority of the paralog pairs populating the two first levels of the functional scale of divergence based on interactions (behaviors I and II) evolved functionally according to the duplication-degeneration-complementation (DDC) or subfunctionalization model proposed by Force *et al*. [28]. This predicts that duplicated genes are preserved by the partitioning of the function(s) of the ancestral gene between the two duplicates. This may happen, for instance, by the complementary loss of regulatory elements or the modification of the coding regions. Even though our analysis does not pretend to reveal the molecular mechanisms by which the subfunctionalization of the duplicated pairs has occurred, several lines of evidence sustain our proposal. First, the first level of the functional scale is populated by paralog pairs, which have kept their interactors identical or still share common interactors. This is in good agreement with a situation in which duplicates have slightly diverged by subfunctionalization to form two subunits of a same complex (for example, Tif4631/Tif4632, Rfc3/Rfc4, Yck1/Yck2; Table 1) or to increase the complexity of a signaling pathway (for instance, Mkk1/Mkk2; Table 1). Second, the intermediate level of the functional scale of divergence (behavior II) contains paralog pairs that do not have the same interactors but have still conserved their cellular function(s) since the duplication event. They may represent paralog pairs involved in different aspects of the same biological process (see Results and [11]) and/or pairs for which the spatio-temporal regulation has evolved by subfunctionalization, therefore implying a new cast of interactors.

Finally, the third level of the functional scale (behavior III) may correspond to duplicates that have evolved by neofunctionalization, as not only their interactors are different but they are also involved in different cellular processes (for instance, Swi5/Ace2). These genes may illustrate Ohno's theory [3] of the emergence of new functions from gene duplication events. Even though we have shown here that there is no simple relationship between sequence identity and cellular function, it is interesting to note that data newly generated by Kellis *et al*. [8] strengthen our proposal. Indeed, the frequency of pairs showing accelerated protein evolution is almost twice as high among the paralog pairs displaying behavior III (37.5% (3/8) of the pairs common to both studies) than among pairs with the same function (20% (5/25) of the pairs common to both studies with behaviors I and II). Overall, these results corroborated our proposal.

## Conclusions

Most network analyses carried out up to now either emphasized the prediction of function for uncharacterized proteins [29,30] or, in the frame of evolutionary studies, estimated the rate of evolution of proteins according to their number of interactors [31] and addressed the issue of the link between protein dispensability and rate of protein evolution [32,33]. As far as we know, this work constitutes the first attempt to address the functional evolutionary fate of duplicated genes using a bioinformatic analysis of the protein-protein interaction network in which the products of these genes are involved, and to provide detailed protein function comparisons based on interaction data. Our approach might thus provide a new way to analyze the evolution of the function of duplicated genes in different organisms.

A limitation of this type of analysis is the present knowledge of interaction networks. Even in a well-studied organism such as *Saccharomyces cerevisiae*, less than 10% of the gene pairs, remnants of the WGD, are amenable to such a detailed analysis. As our knowledge on interaction networks is increasing and as more interactions become available, we can expect to improve both the coverage of duplicated pairs of interactors and the relevance of the functional clusters found by the PRODISTIN method.

Finally, it should be emphasized that the study of evolutionary processes greatly benefits by being approached using different tools not only at the sequence level, as is usual, but also directly at the functional level. In the case of the study of the 41 paralog pairs reported here, functional conclusions inferred from the sequence level would have been incomplete and even erroneous in several instances.

## Materials and methods

### Functional distance based on GO annotations

GOproxy [13], a tool that calculates the Czekanowski-Dice distance between gene annotations was used to compare the GO annotations [12] of the duplicated gene products as well as that of five datasets of 460 pairs of proteins randomly selected from the yeast genome. The Molecular Function, Biological Process and Cellular Component ontologies were processed separately. The Czekanowski-Dice distance formula used in the algorithm is:

Dist(*i,j*) = number of (Terms(*i*) ΔTerms(*j*))/ [number of (Terms(*i*) ∪ Terms(*j*)) + number of (Terms(*i*) ∩ Terms(*j*))],

in which, *i *and *j *denote two genes, Terms(*i*) and Terms(*j*) are the lists of their GO terms and Δ is the symmetrical difference between the two sets. This distance formula increases the weight of the shared GO terms by giving more weight to similarities than to differences. The GOToolBox website can be accessed at [13].

### Protein-protein interaction dataset

The protein-protein interaction dataset we investigated contains a total of 4,143 selected interactions involving 2,643 proteins. We updated our former dataset [11] with 1,244 new interactions taken from the Munich Information Center for Protein Sequences (MIPS) [34] and from the literature. As previously, only direct binary interactions were selected according to the method used for their identification (two-hybrid experiments, *in vitro *binding, far western, gel retardation and biochemical experiments).

### PRODISTIN analysis

PRODISTIN, a computational method we recently proposed [11], was used to analyze the protein-protein interaction dataset. Starting with a binary list of interactions, only proteins involved in at least three binary interactions were selected for further classification (because poorly connected proteins have a higher chance of being involved in false-positive interactions). A graph in which vertices are proteins and edges correspond to the relation 'interact with and/or share at least one common interactor' was computed and the Czekanowski-Dice distance was calculated between all possible pairs of proteins belonging to the connected component of this graph (using the formula above and applying it to the list of protein interactors instead of the list of GO terms). The distance matrix was then clustered using BioNJ [35] and the tree was visualized using TreeDyn [36]. PRODISTIN classes corresponding to the largest possible subtree composed of at least three proteins sharing the same functional annotation and representing at least 50% of the individual class members for which a functional annotation is available were detected in the tree. GO annotations corresponding to the Biological Process ontology were used for this purpose. Given that GO is organized as a DAG, proteins may be annotated at different levels of the ontology. Our goal was to analyze subtrees regarding to the proteins commonly annotated as participating in them, so we considered annotations for all proteins at a specific level of the ontology. We chose to work at level 4 because we estimated, on previous experience using the Yeast Proteome Database [37] system of annotation, that this particular level provides a good representation of the complexity of cellular functions. For this, we used GODiet, a tool enabling us to restrict the list of GO terms to a given depth in the ontology [13].

### Sequence analysis

Pairwise sequence alignments were carried out on the set of 460 pairs of duplicated protein sequences using the Needleman-Wunsch (global alignment) algorithm. The program used is available at [38]. The chosen alignment matrix was BLOSUM50, and the gap-opening and gap-extension penalties were set to 12 and 2, respectively. The resulting 460 alignments have been processed to calculate the percent identity for each protein pair.

## Additional data files

The following additional data are available with the online version of this paper. Additional data file 1 contains the expectation values for the distribution of functional distances based on the GO annotations. Additional data file 2 contains details of the 123 PRODISTIN classes contained in the classification tree.

## Supplementary Material

Additional data file 1 The expectation values for the distribution of functional distances based on the GO annotations Click here for additional data file

Additional data file 2 Details of the 123 PRODISTIN classes contained in the classification tree Click here for additional data file

## Figures and Tables

**Table 1:** Details of the behaviors of the 41 duplicated pairs present in the PRODISTIN classification tree

Behavior class	Gene 1	Gene 2	Localization in same PRODSTIN class	Same cellular function	Annotation of the PRODISTIN classes by cellular function
I	ARF1	ARF2	+	+	Vesicle-mediated transport, secretory pathway, intracellular transport (50)
	ASM4	NUP53	+	+	Nuclear organization and biogenesis (22), nucleobase nucleoside nucleotide and nucleic acid transport, protein targeting, RNA localization (32), nucleobase nucleoside nucleotide and nucleic acid metabolism, intracellular transport (48)
	BMH2	BMH1	+	+	Energy derivation by oxidation of organic compounds, polysaccharide metabolism, carbohydrate metabolism (6)
	BOI1	BOI2	+	+	Nuclear organization and biogenesis (22), nucleobase nucleoside nucleotide and nucleic acid transport, protein targeting, RNA localization (32), nucleobase nucleoside nucleotide and nucleic acid metabolism, intracellular transport (48)
	ECI1	DCI1	+	+	Cytoplasm organization and biogenesis, protein targeting (7)
	GIC2	GIC1	+	+	Bud growth (6), intracellular signaling cascade (26), signal transduction (58), cytoplasm organization and biogenesis (94)
	GZF3	DAL80	+	+	Transcription, nitrogen utilization (5), nucleobase nucleoside nucleotide and nucleic acid metabolism (66)
	KCC4	GIN4	+	+	Cell cycle(16), nucleobase nucleoside nucleotide and nucleic acid metabolism, intracellular transport (48)
	MKK1	MKK2	+	+	Phosphate metabolism, protein modification (6), conjugation with cellular fusion, sensory perception, perception of abiotic stimulus (20), signal transduction (58), cytoplasm organization and biogenesis (94)
	MYO3	MYO5	+	+	Polar budding, vesicle-mediated transport, response to osmotic stress (5), cytoplasm organization and biogenesis (10), nucleobase nucleoside nucleotide and nucleic acid metabolism (55)
	NUP100	NUP116	+	+	Nuclear organization and biogenesis (22), nucleobase nucleoside nucleotide and nucleic acid transport, protein targeting, RNA localization(32), nucleobase nucleoside nucleotide and nucleic acid metabolism, intracellular transport (48)
	PCL6	PCL7	+	+	Energy derivation by oxidation of organic compounds, polysaccharide metabolism, carbohydrate metabolism (5), transcription (17)
	RAS2	RAS1	+	+	Intracellular signaling cascade(4), cell proliferation (20)
	RFC3	RFC4	+	+	DNA repair, response to DNA damage stimulus, cell cycle(18), nucleobase nucleoside nucleotide and nucleic acid metabolism (23)
	SEC4	YPT7	+	+	Vesicle-mediated transport, secretory pathway, intracellular transport (50)
	SIZ1	NFI1	+	+	External encapsulating structure organization and biogenesis, cell proliferation, cellular morphogenesis (8), signal transduction (58), cytoplasm organization and biogenesis (94)
	SSK22	SSK2	+	+	Phosphate metabolism, intracellular signaling cascade, protein modification (5), cell surface receptor linked signal transduction nucleobase nucleoside, nucleotide and nucleic acid metabolism (7)
	SSO2	SSO1	+	+	Vesicle-mediated transport (14)
	TIF4632	TIF4631	+	+	Protein biosynthesis (7), macromolecule biosynthesis (12), nucleobase nucleoside nucleotide and nucleic acid metabolism (55)
	VPS64	YLR238W	+	+	Response to pheromone during conjugation with cellular fusion, sensory perception, perception of abiotic stimulus (6), cell cycle, cytoplasm organization and biogenesis (16)
	YIL105C	YNL047C	+	+	Unknown (4)
	YPT31	YPT32	+	+	Vesicle-mediated transport, secretory pathway, intracellular transport (50)
	YPT53	VPS21	+	+	Cytoplasm organization and biogenesis (6), vesicle-mediated transport, secretory pathway, intracellular transport (50)
	ZDS2	ZDS1	+	+	Cell aging, response to DNA damage stimulus, chromatin silencing(5), intracellular signaling cascade (26), cytoplasm organization and biogenesis (94), signal transduction (58)
	RPS26B	RPS26A	+	+	Nucleobase, nucleoside, nucleotide and nucleic acid metabolism (29)
	YCK1	YCK2	+	+	Transport (6), nucleobase, nucleoside, nucleotide and nucleic acid metabolism (202)
					
II	BUB1	MAD3	-	+	Cell cycle, cell proliferation (40), nucleobase, nucleoside, nucleotide and nucleic acid metabolism (66)
	TUB4	TUB1	-	+	Cell cycle, cytoplasm organization and biogenesis (16)
					Cell cycle, cytoplasm organization and biogenesis (7)
	ENT1	ENT2	-	+	Cytokinesis, vesicle-mediated transport, cytoplasm organization and biogenesis (4), cell proliferation (20)
					Vesicle-mediated transport (14)
					
III	YAP1802	YAP1801	-	-	Cell proliferation (20)
					Vesicle-mediated transport (14)
	YMR181C	YPL229W	-	-	Cell proliferation (20)
					Transcription (8), nucleobase, nucleoside, nucleotide and nucleic acid metabolism (202)
	NUP170	NUP157	-	-	Nuclear organization and biogenesis (22), nucleobase nucleoside nucleotide and nucleic acid transport, protein targeting, RNA localization (32), nucleobase nucleoside nucleotide and nucleic acid metabolism, intracellular transport (48)
					Cell cycle, cytoplasm organization and biogenesis (7)
	APP2	GYP5	-	-	Vesicle-mediated transport (18), transport (21), cytoplasm organization and biogenesis (94)
					RNA metabolism (29), nucleobase, nucleoside, nucleotide and nucleic acid metabolism (202)
	SIR2	HST1	-	-	Cell cycle, chromatin silencing(6), nucleobase, nucleoside, nucleotide and nucleic acid metabolism (14)
					RNA metabolism (9), nucleobase, nucleoside, nucleotide and nucleic acid metabolism (202)
	GSP1	GSP2	-	-	Nuclear organization and biogenesis (22), nucleobase, nucleoside, nucleotide and nucleic acid transport, protein targeting, RNA localization(32), nucleobase, nucleoside, nucleotide and nucleic acid metabolism, intracellular transport (48)
					Cell cycle (4)
	SWI5	ACE2	-	-	Transcription (6), macromolecule biosynthesis (11), nucleobase, nucleoside, nucleotide and nucleic acid metabolism (55)
					Cell cycle (4)
	LSB1	PIN3	-	-	Unknown (5), nucleobase, nucleoside, nucleotide and nucleic acid metabolism (23)
					RNA metabolism (29), nucleobase, nucleoside, nucleotide and nucleic acid metabolism (202)
	YBR270C	BIT61	-	-	Unknown (4)
					Transport (21), cytoplasm organization and biogenesis (94)
					
NC	EBS1	EST1			
	MTH1	STD1			
	NMA2	NMA1			

+ and - indicate the status of the duplicates in respect of their localization in the same PRODISTIN class and whether they have the same cellular functions. NC, not classified, indicating the pairs for which at least one of the genes does not belong to a PRODISTIN class. The last column shows the annotation of the PRODISTIN classes containing the duplicated genes and the number of class members (in parentheses). When the 2 genes of the pair belong to different classes (behavior II and III), the first list of annotations corresponds to the class containing gene 1 and the second list to the one containing gene 2.

**Table 2:** Summary of the behaviors of the 41 duplicated genes

Classification behaviors	Number of duplicated pairs
I Same class, same biological process	26 (63%)
II Different classes, same biological process	3 (7.5%)
III Different classes, different biological process	9 (22%)
Not classified	3 (7.5%)
Total	41
