# Supplementary material for: A scale of functional divergence for yeast duplicated genes revealed from analysis of the protein-protein interaction network
Source: Genome Biol. 2004 Sep 15;5(10):R76. doi: 10.1186/gb-2004-5-10-r76 (PMC545596; doi:10.1186/gb-2004-5-10-r76)
Supplement: Additional data file 1 — The expectation values for the distribution of functional distances based on the GO annotations [file gb-2004-5-10-r76-s1.doc]

Additional data file 1: Expectation values for the distribution of functional distances based on the GO annotations

|  | Molecular Function | Biological Process | Cellular Component |
| --- | --- | --- | --- |
| Random pairs | 0.90 | 0.78 | 0.52 |
| Paralogue pairs | 0.09 | 0.16 | 0.14 |
